# A cartilage-forming tumor of the mandibular angle: a case report 

**DOI:** 10.1186/s13256-022-03359-x

**Published:** 2022-04-28

**Authors:** Ayman Ismail, Imane Boujguenna, Koussay Hattab, Nadia Mansouri, Najat Cherif Idrissi El Ganouni, Mariem Ouali Idrissi, Fatima Ezzahra Hazmiri, Hanane Rais

**Affiliations:** 1 Department of Pathology and Biopathology Unit, Clinical Research Center, MOHAMMED VI University Hospital of Marrakech, Faculty of Medicine and Pharmacy of Marrakech, Cadi Ayyad University of Marrakech, Marrakech, Morocco; 2 Department of Oral and Maxillofacial surgery, MOHAMMED VI University Hospital of Marrakech, Faculty of Medicine and Pharmacy of Marrakech, Cadi Ayyad University of Marrakech, Marrakech, Morocco; 3 Department of Radiology, MOHAMMED VI University Hospital of Marrakech, Faculty of Medicine and Pharmacy of Marrakech, Cadi Ayyad University of Marrakech, Marrakech, Morocco; 4 Laboratory of Histology and Embryology, Department of Preclinical Science, Faculty of Medicine and Pharmacy of Marrakech, Cadi Ayyad University of Marrakech, Marrakech, Morocco

**Keywords:** Cartilage-forming tumor, Mandible, Chondrosarcoma, Evan’s grading system, Chondroblastic osteosarcoma, Mandibulectomy

## Abstract

**Background:**

Mandible can be the site of benign or malignant lesions of different origins, including odontogenic and non-odontogenic lesions. Cartilage-forming tumors have been rarely reported at this site. Chondrosarcoma is a rare malignant cartilage-producing neoplasm that is extremely rare in the mandible. The rarity of cartilage-forming tumor occurrence in the mandible can make diagnosis difficult for pathologists, as they do not expect this type of tumor at this anatomical site. Here we report a case of chondrosarcoma of mandibular angle.

**Case presentation:**

A 70-year-old Moroccan male patient consulted a dentist for wisdom tooth pain. Wisdom tooth extraction was conducted. After 6 months, the patient reported the recurrence of pain associated with swelling in the mandibular area and paresthesia along the path of the mandibular nerve. A panoramic radiograph demonstrated a mixed radiolucent–opaque lesion involving the mandibular angle. Computed tomography showed a large osteolytic spontaneously hypointense and multilobulated lesion. A biopsy was done. Histopathological examination revealed sheets and irregular lobules of atypical cells presenting cartilaginous differentiation. Tumor cells showed severe nuclear atypia and were located within a hyaline cartilage matrix. Some foci of necrosis were noted. Osteoid deposits were not found. The patient was diagnosed with grade III chondrosarcoma and underwent a right segmental mandibulectomy with submandibular lymph node dissection. Macroscopically, the tumor was localized in the mandibular angle with extension in the mandibular body. Histopathology confirmed the previous diagnosis of grade III chondrosarcoma and did not show any lymph node metastasis.

**Conclusions:**

Owing to many histological similarities, grade III chondrosarcoma must be distinguished from chondroblastic osteosarcoma and metastatic lesions. In addition, chondroblastic osteosarcoma of the jawbones has a worse prognosis than chondrosarcoma, making the distinction between these two malignant tumors the most important concern of the pathologist when dealing with a cartilage-forming tumor at this site. Surgery with wide excision margins remains the best therapeutic approach, while the role of radiotherapy is controversial. The management of mandibular chondrosarcoma requires a multidisciplinary approach involving maxillofacial surgeons, radiologists, pathologists, and oncologists.

## Background

Mandible can be the site of various lesions that can be of odontogenic or non-odontogenic origin [1]. The most frequent tumors occurring in the mandible are odontogenic tumors dominated by keratocystic odontogenic tumors and ameloblastoma [2, 3]. Non-odontogenic malignant tumors remain very rare, the most frequent ones being squamous cell carcinoma, osteosarcoma, and metastatic lesions [1]. Cartilage-forming tumors have been rarely reported in the mandible [1]. Chondrosarcoma is a rare slowly growing malignant tumor characterized by cartilage production and composed of chondrocytes with variable degrees of malignancy [4]. Chondrosarcoma represents a group of locally aggressive or malignant tumors producing cartilage matrix without tumor osteoid deposits [5]. Chondrosarcoma is the second most common type of primary bone cancer after osteosarcoma [4]. It most commonly affects flat bones, such as the ilium and the scapula, and can also occur in the appendicular skeleton, where the femur and the tibia are most often involved [6]. The location at the maxillofacial region is extremely rare, accounting for less than 3% of the entire body [4]. In this anatomical region, the most common site of occurrence remains the maxillary bone. The mandible is less frequently involved [7]. The rarity of cartilage-forming tumor occurrence in the maxillofacial bones increases the risk of misdiagnosis, considering that pathologists do not expect these tumor types at those anatomical sites [8].

## Case presentation

A 70-year-old Moroccan male patient with unremarkable personal and familial medical history consulted a dentist for wisdom tooth pain lasting for 2 months. A wisdom tooth extraction was conducted. After 6 months, the patient reported the recurrence of pain in the wisdom tooth area occurring at rest and while chewing associated with paresthesia along the path of the mandibular nerve. In addition, the patient complained of a swelling on the right side of the mandibular area. The patient was referred to the oral and maxillofacial surgery department of our hospital. Intraoral examination revealed a well-defined nodular mass located at the junction of the mandibular angle and the body of the mandible. The overlying mucosa showed no abnormalities. Extraoral examination showed multiple submandibular lymph nodes.

**Fig. 1:**
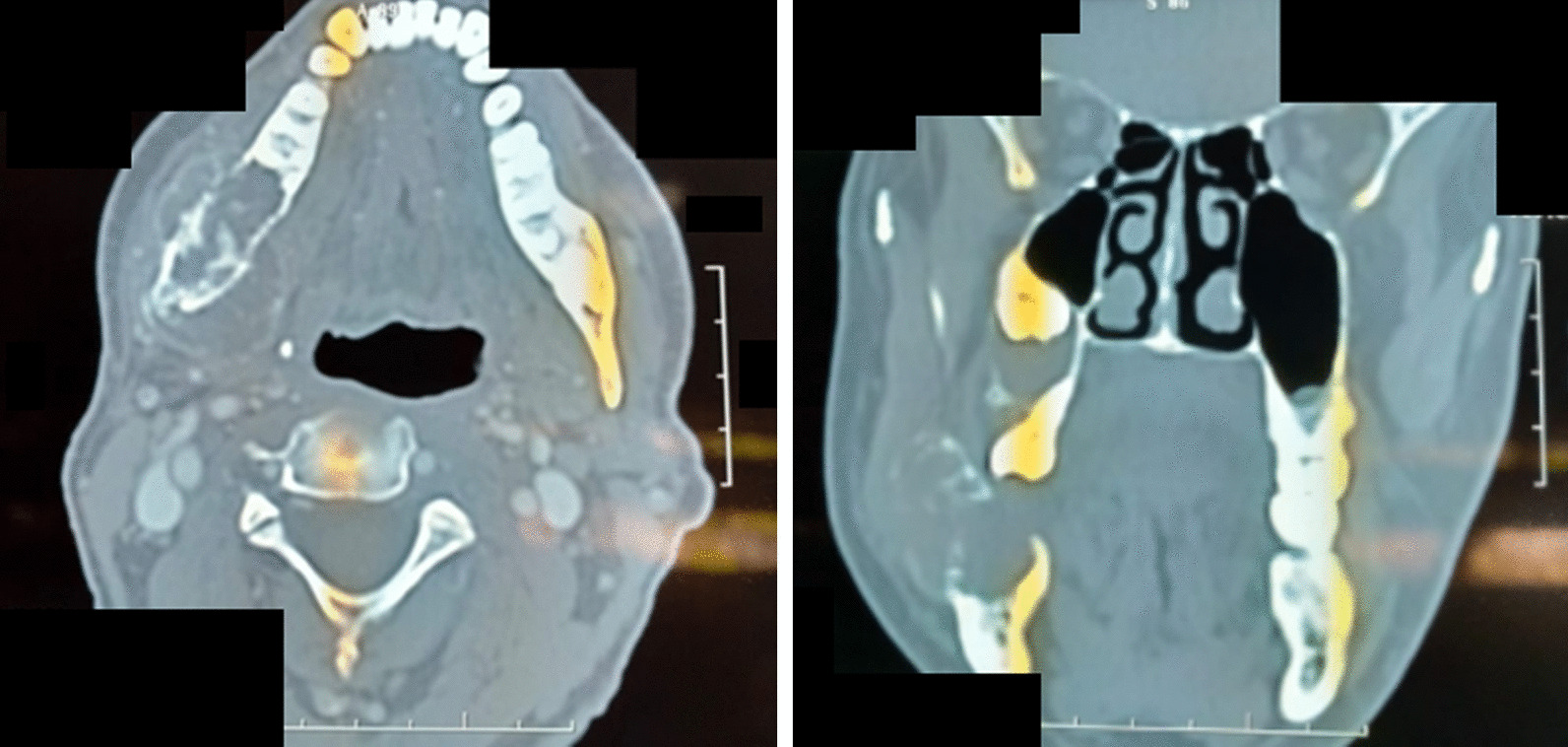
Computed tomography scan showing a large osteolytic spontaneously hypointense and multilobulated lesion associated with calcified areas with extension to the masseter muscle and the anterior belly of digastric muscle

A panoramic radiograph revealed a mixed radiolucent–opaque lesion involving the mandibular angle and associated with the displacement of tooth 18. Computed tomography (CT) demonstrated a large osteolytic spontaneously hypointense and multilobulated lesion associated with calcified areas measuring 47 mm in the largest diameter with extension to the masseter muscle and the anterior belly of digastric muscle (Fig. 1).

**Fig. 2:**
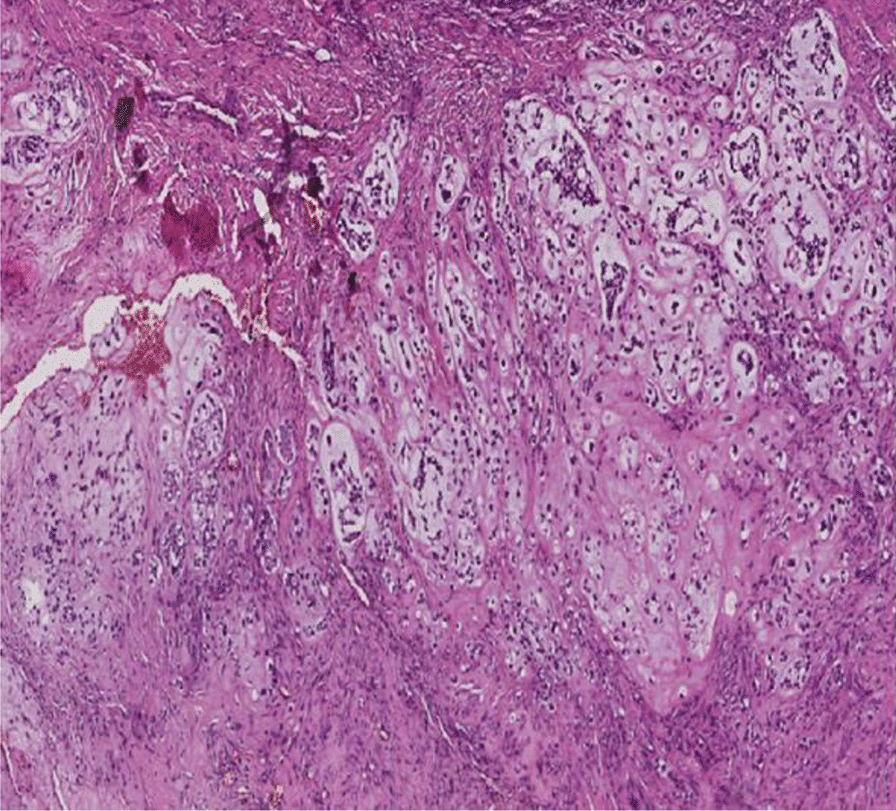
Tumor composed of sheets and irregular lobules of atypical cells presenting cartilaginous differentiation

**Fig. 3:**
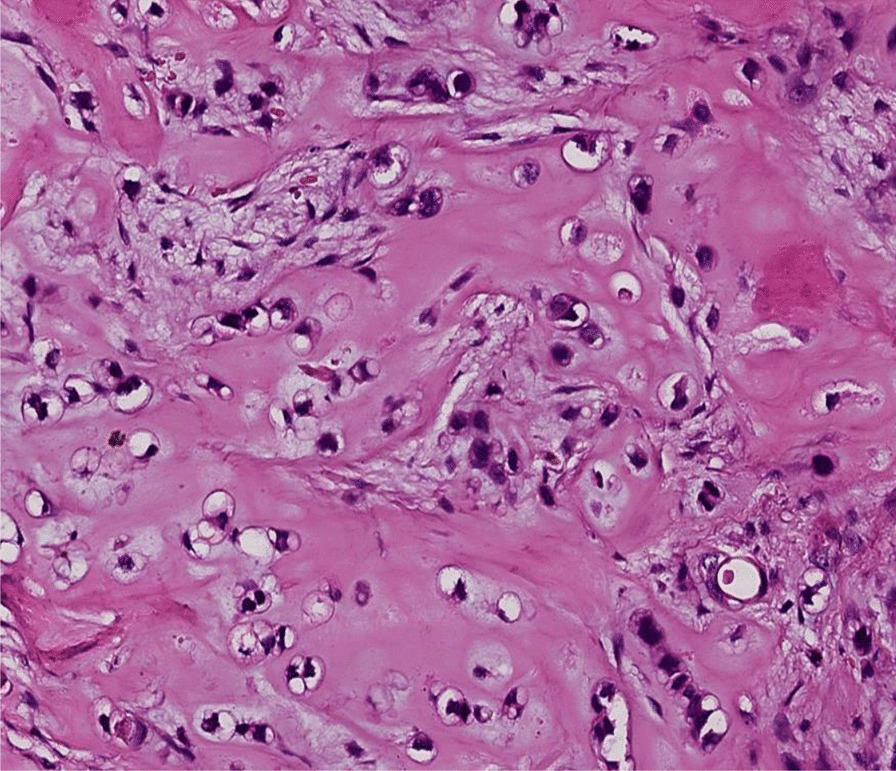
Tumor cells showing nuclear enlargement, hyperchromasia, focal binucleation, and partial loss of lacunar arrangement

Thereafter, an incisional biopsy was done. On gross examination, the specimens were represented by five whitish tissue fragments firm in consistency, the largest of which measured 1.7 cm. Microscopically, the tumor was composed of sheets and irregular lobules of atypical cells presenting cartilaginous differentiation (Fig. 2). Tumor cells were enlarged with partial loss of lacunar arrangement and severe nuclear atypia consisting of nuclear enlargement, hyperchromasia, and focal binucleation (Fig. 3). The mitosis count was three mitoses per ten high-power fields (HPF). Tumor lobules were separated by thick fibrous bands and showed in their periphery oval and spindle dedifferentiated cells. The stroma was made of a hyaline chondroid matrix associated with myxoid areas without evidence of malignant osteoid. Some foci of necrosis were noted. The patient’s history, clinical, and imaging features in addition to histopathological findings suggested an osseous malignant tumor process. The presence of extensive areas of chondroid differentiation raised the diagnosis of chondroblastic osteosarcoma and chondrosarcoma. The presence of punctate intraosseous calcifications on CT and the absence of osteoid deposit after careful examination were consistent with the diagnostic of chondrosarcoma. The grading was established according to the Evans Grading system, which is based on the degree of cellularity, cytological atypia, and mitosis. In the present case, histopathological examination demonstrated high cellularity within sheets and tumor lobules, with tumor cells harboring severe atypia, partial loss of lacunar arrangement, and a mitosis count of more than two mitoses per ten HPF in addition to myxoid stromal change. These histopathological findings were suggestive of a grade III chondrosarcoma.

**Fig. 4:**
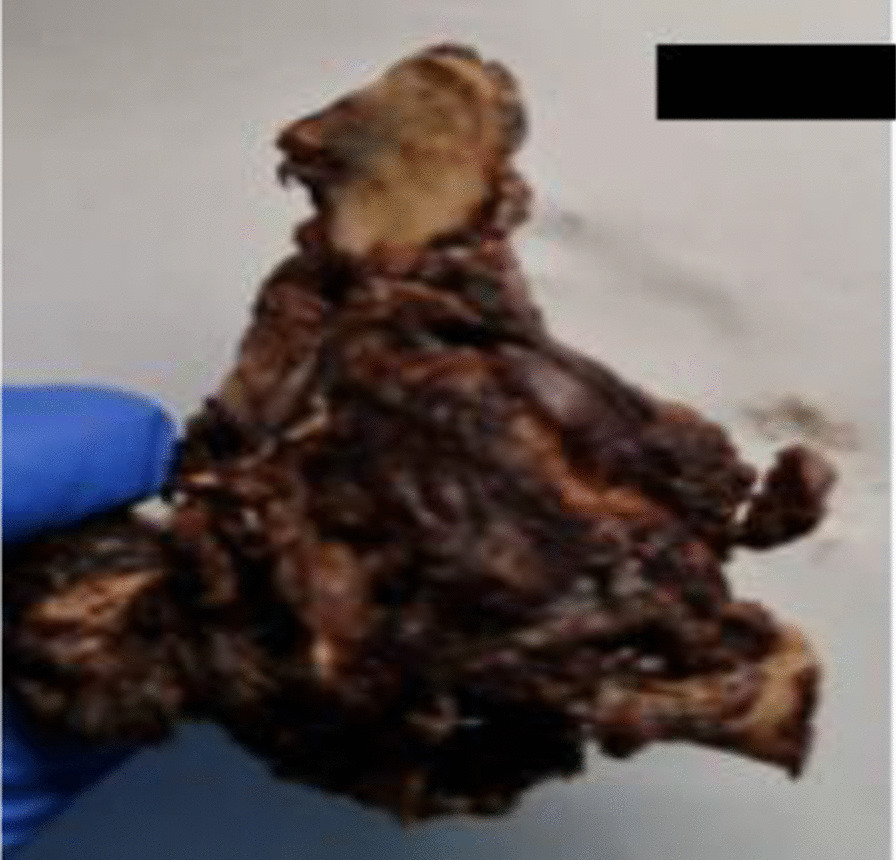
Right segmental mandibulectomy showing a tumor resection from tooth 20 to the coronoid process

**Fig. 5:**
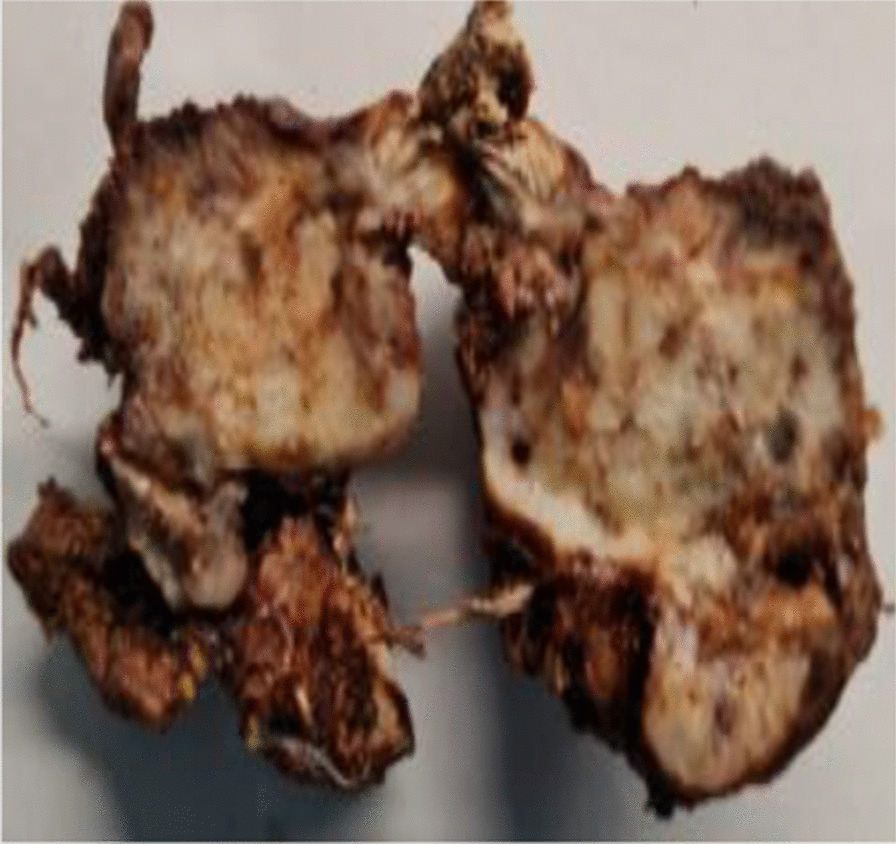
Section slice showing a multilobulated whitish tumor measuring 4.5 × 2.8 × 2 cm

**Fig. 6:**
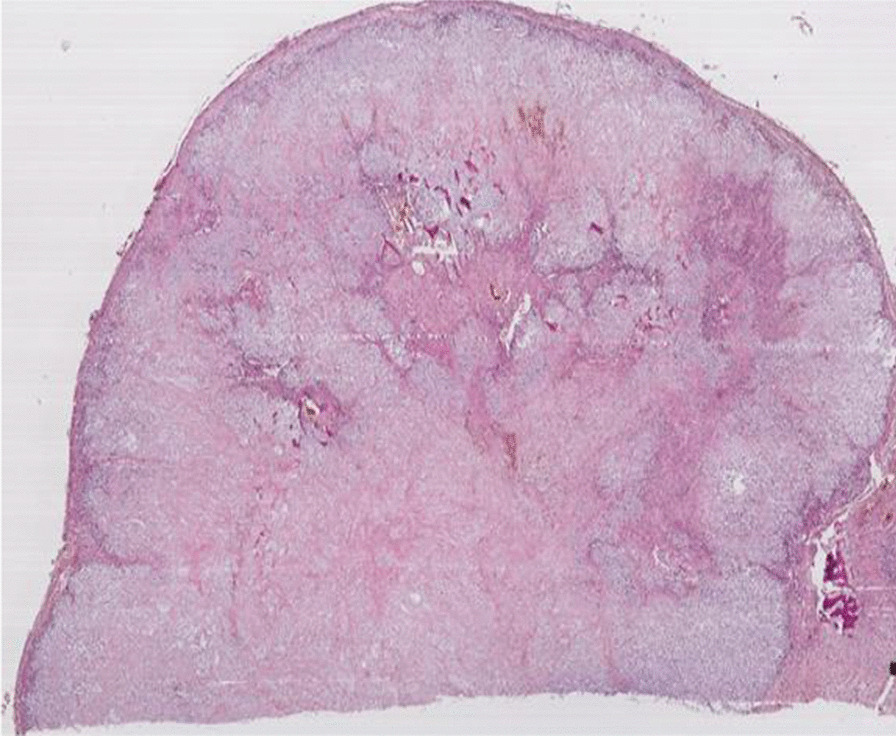
Tumor growth delimited by a thin fibrous capsule (magnification ×4)

**Fig. 7:**
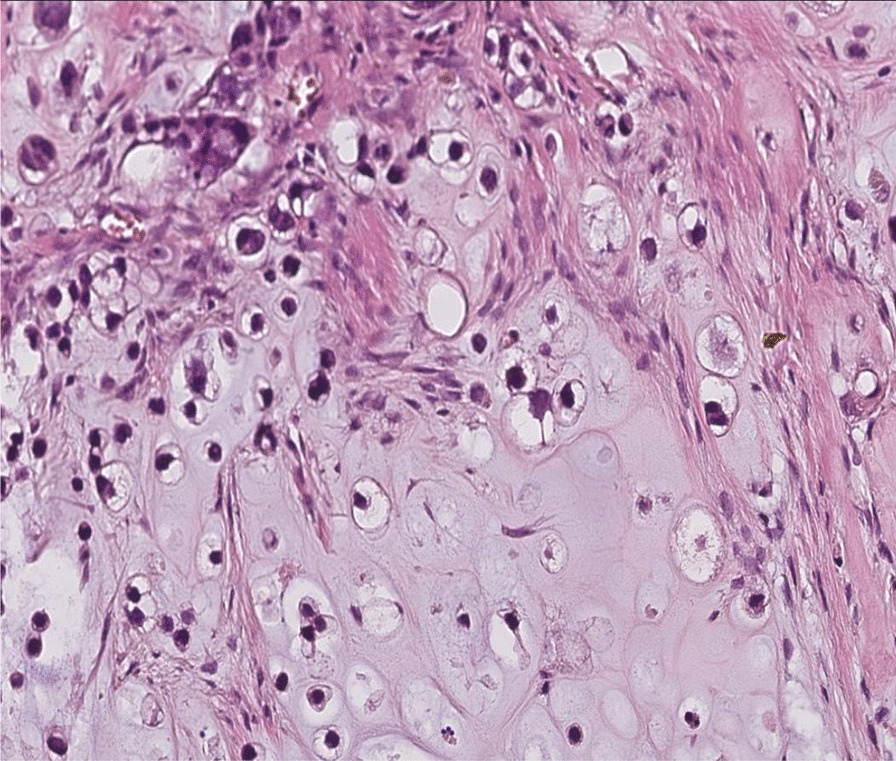
Sheets and lobules of atypical cells separated by thick fibrous bands

**Fig. 8:**
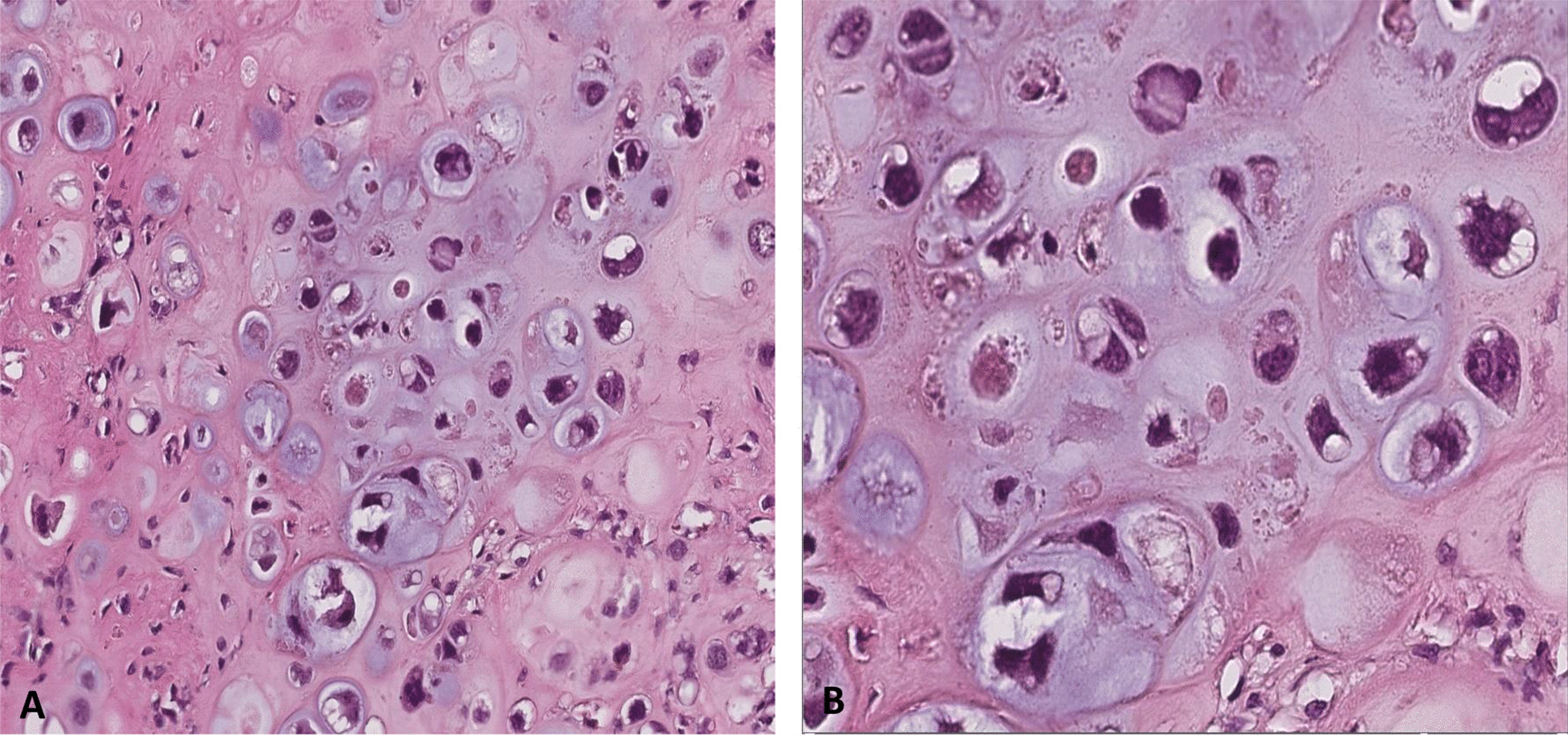
Atypical cells within abundant cartilage matrix exhibiting marked nuclear atypia consisting of nucleomegaly, hyperchromasia, irregular contours, binucleation, abnormal mitosis, and partial loss of lacunar arrangement (**A** magnification ×20, **B** magnification ×40)

**Fig. 9:**
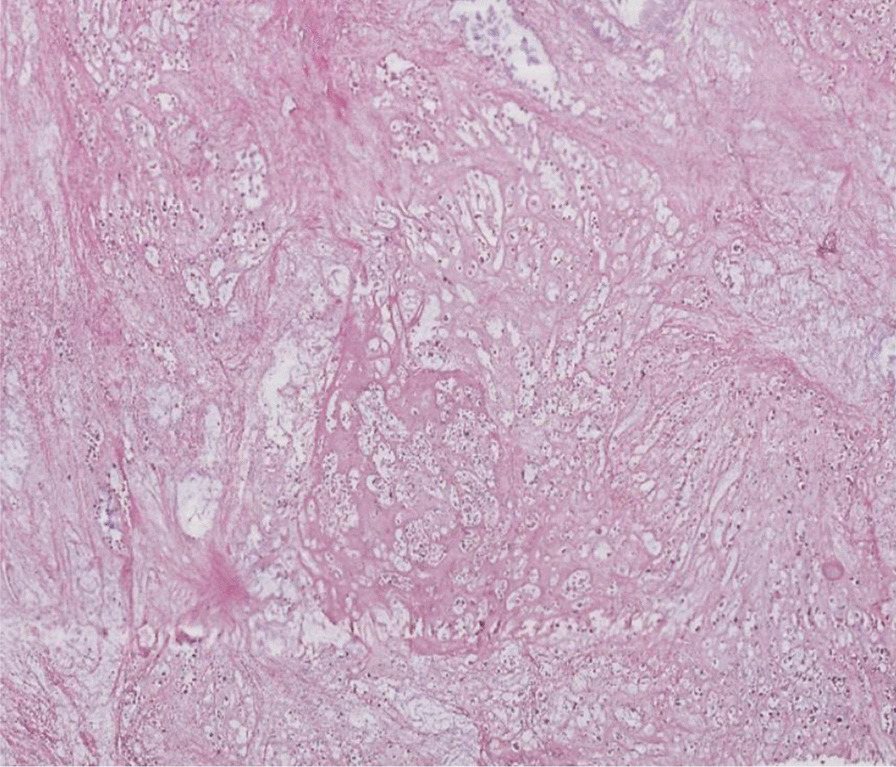
Extensive areas of necrosis

The patient underwent surgery, which consisted of a right segmental mandibulectomy (Fig. 4). The tumor was resected from tooth 20 to the coronoid process with reconstruction by titanium osteosynthesis angled plate and submandibular lymph node dissection. Macroscopically, the tumor was localized in the mandibular angle with extension in the mandibular body opposite tooth 19 and extension in the mandibular ramus. The tumor measured 4.5 × 2.8 × 2 cm, and it was whitish, multilobulated, firm in consistency (Fig. 5), encapsulated at the internal side, and infiltrating the masticatory muscles at the external side. Osseous and muscular resection margins were macroscopically free of the tumor. Dissection of the submandibular area revealed eight indurated lymph nodes measuring between 0.5 and 1.6 cm. Microscopic examination showed a tumor growth delimited by a thin fibrous capsule (Fig. 6) displaying sheets and lobules of atypical cells separated by thick fibrous bands (Fig. 7). The latter exhibited marked nuclear atypia consisting of nucleomegaly, hyperchromasia, irregular contours, binucleation, abnormal mitosis, and partial loss of lacunar arrangement (Fig. 8). Hyaline cartilage matrix was abundant (Fig. 9). Tumor cells at the periphery of the lobules were dedifferentiated harboring a spindled morphology. Extensive areas of necrosis were noted (Fig. 9). After exhaustive sampling, no osteoid deposit was identified, thus confirming the previous diagnosis of grade III chondrosarcoma. The resection margins were free of the tumor. No submandibular lymph nodes harbored the tumor. The tumor stage was pT1N0Mx according to the AJCC 8th edition. After surgery, a slight limitation of mouth opening was reported by the patient. No aesthetic damage was noted.

**Fig. 10:**
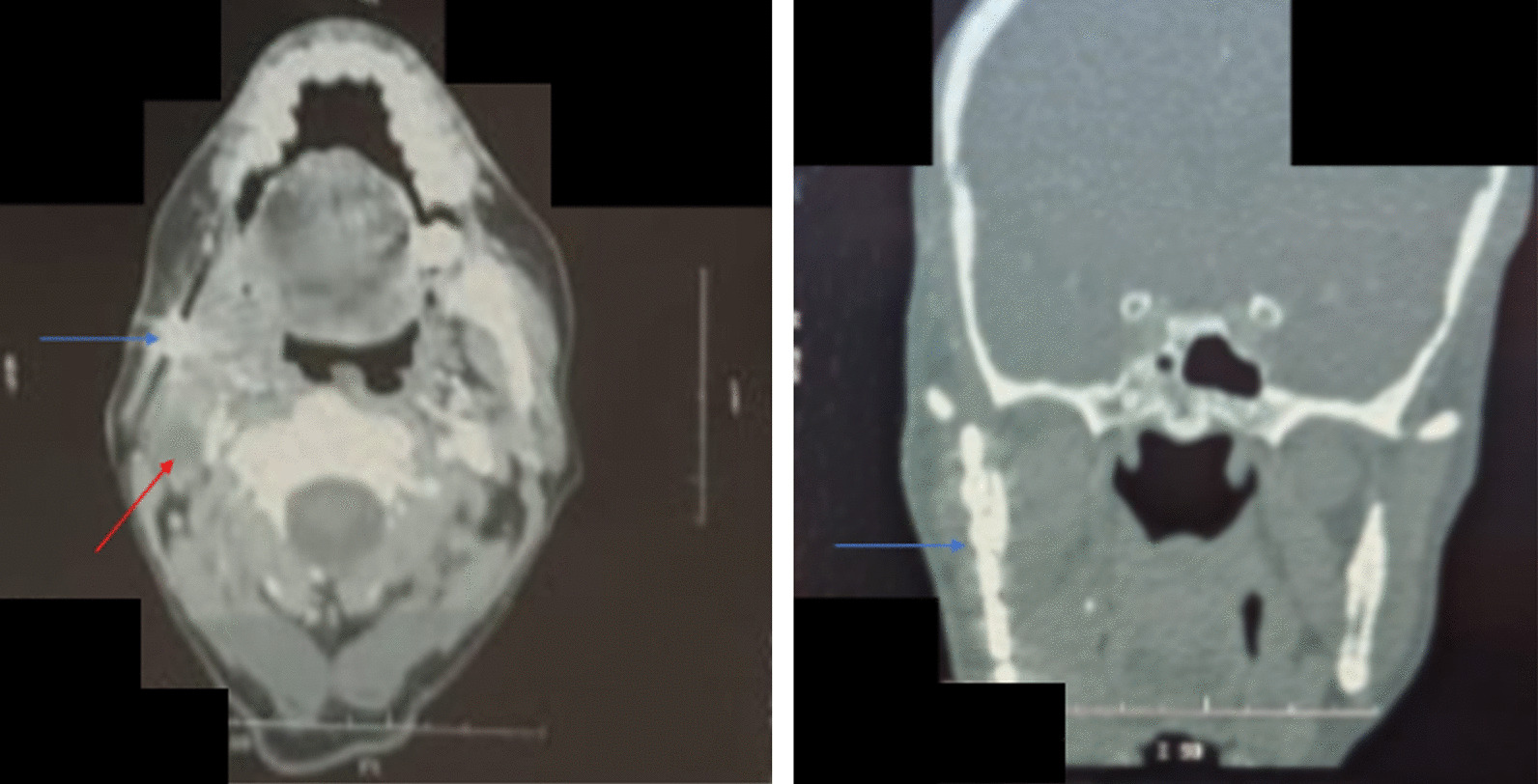
CT scan showing jugulocarotid lymphadenopathy measuring 33 × 15 mm with a necrotic center (red arrow) and angled reconstruction plate (blue arrow)

**Fig. 11:**
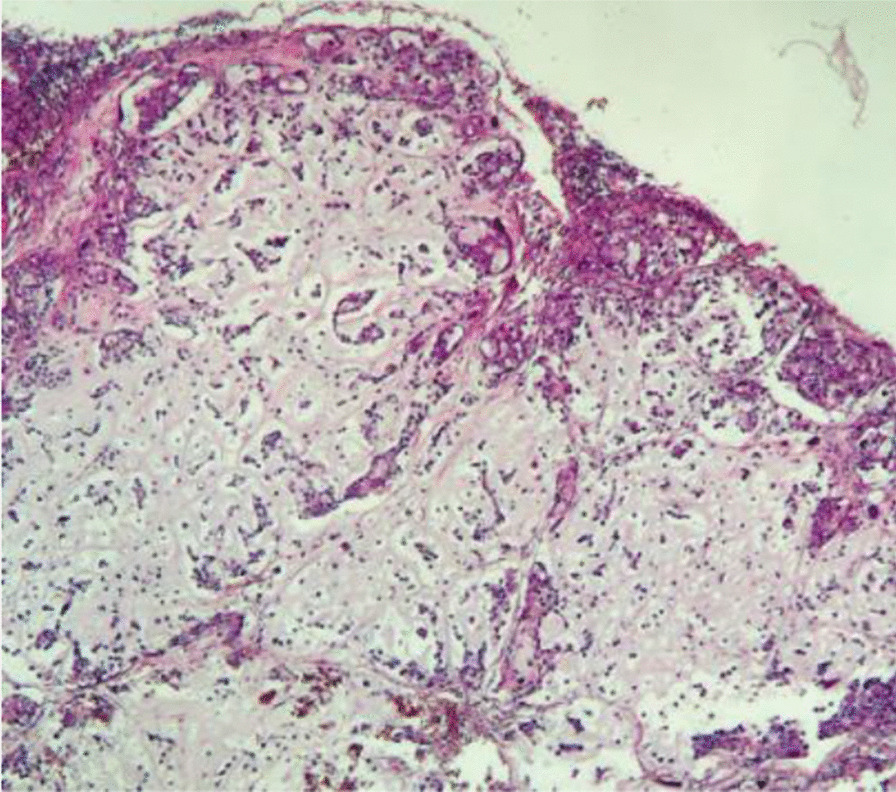
Lymph node parenchyma completely involved in a tumor growth similar to the one previously diagnosed

Four months later, the patient presented under the incision area a firm swelling. A CT was done and showed a jugulocarotid lymphadenopathy measuring 33 × 15 mm with a necrotic center (Fig. 10). A surgical resection was performed. Histopathological examination of the resected specimen revealed a lymph node parenchyma completely involved in a tumor growth similar to the one previously diagnosed, confirming a recurrence (Fig. 11). After a multidisciplinary meeting with participation from maxillofacial surgeons, radiologists, oncologists, and radiation therapists, a complementary treatment by radiation therapy was decided for the patient with total irradiated dose of 60 Gy in 30 fractions. No severe radiation-related complications were reported during treatment. No adverse events were recorded. The patient is currently under clinical follow-up without any evidence of recurrence.

## Discussion

The chondrosarcoma is a malignancy that produces cartilage matrix and occurs exceptionally in the bones of oral and maxillofacial region, accounting for 1–3% of all chondrosarcomas [9, 10]. At this anatomical site, the involvement of the anterior maxillary region is predominant where preexisting nasal cartilage is present, while it rarely occurs in the mandible [10]. Most mandibular chondrosarcomas arise from the molar and the symphyseal region and they exceptionally develop in the ramus, condyle, or coronoid process [4, 10].

### Clinical features

Chondrosarcomas of the jaw tend to occur equally in both sexes [9]. The most affected age range is between the third and sixth decades of life [11]. The main manifestation of jaws chondrosarcomas is painless swelling or enlarging mass evolving for a long period that may extend to buccal and lingual cortical plates with the possibility of teeth exfoliation [4, 9, 10]. Dental complaints may occur as an initial symptom [9]. Other symptoms include displacement of involved teeth and widening of the parodontium [12]. Pain, paresthesia, trismus, and loosening of the teeth may be related to the progression of the disease [7].

### Imaging findings

Radiographically, the lesion appearance is not pathognomonic; nevertheless, it most commonly appears radiolucent with ill-defined outlines often associated with evidence of bone destruction [4]. Other lesions may also have the same radiological appearance such as periodontal cysts, odontogenic cysts, odontogenic tumors, and central giant cell granuloma [4]. CT scan allows the detection of matrix mineralization and often exhibits a mass with an inhomogeneously mineralized center that may be associated with adjacent bone destruction; it also provides information on the peripheral extent of the neoplasm [13, 14]. Imaging is very useful for defining the nature and extent of the lesion, but histology determines the definitive diagnosis [7].

### Pathology

On gross examination, conventional intramedullary chondrosarcomas are large tumors often greater than 4 cm in size [13]. The cut surface is gray white with lobulated borders [15]. The consistency is commonly firm but can also be mucoid or gelatinous [15].

Histopathologically, the spectrum of chondrosarcoma varies widely from well-differentiated neoplasms that may be confused with benign cartilaginous tumors to locally aggressive high-grade tumors with metastatic potential [4]. The grading system proposed by Evans is widely used, which is based on cell density, nuclear size and staining, and mitosis [16].

Grade I chondrosarcomas are characterized by a lobular histologic appearance, where lobules can show variation in size and shape [6]. Hyaline cartilage matrix is abundant, and fibrous bands containing small vessels separate the lobules [6]. The cellularity is low, and chondrocytes have small uniform nuclei that might be slightly enlarged [13]. Mitoses are absent [6]. Binucleation is a common feature that may help in the distinction of grade I chondrosarcomas from enchondroma, but bone entrapment and myxoid matrix change seen in grade I chondrosarcomas are more discriminating [6, 13].

Grade II chondrosarcoma show increased cellularity with less chondroid matrix particularly at the periphery of tumor [13, 15]. The lacunar path is usually retained, and myxoid matrix changes are often demonstrated within the stroma [13, 15]. The chondrocyte nuclei vary in size with either vesicular chromatin with visible nucleolus or condensed chromatin [6, 15]. Chondrocytes may be binucleated or multinucleated [13] and rare mitotic figures can be seen [6]. Nuclear atypia is present but still mild to moderate [6]. Tumor necrosis ranging from small foci to completely necrotic lobules can be present [13]. Grade II chondrosarcoma tend to recur locally more than grade I lesion and may metastasize in 10% of cases [4].

In grade III chondrosarcoma, cellularity is high and lobules are composed of tumor cells displaying marked nuclear atypia and pleomorphism with at least at two mitoses found per 10 HPFs [6, 15]. The cells at the periphery of the lobules are usually spindled and less differentiated [6]. The lacunar arrangement is lost, and the intercellular material is mostly myxoid containing a small amount of chondroid matrix that can be totally absent in some cases [13]. Necrosis is generally extensive and almost invariably seen [10]. Owing to many histological similarities, grade III chondrosarcoma must be distinguished from chondroblastic osteosarcoma [4]. In addition, chondroblastic osteosarcoma of the jawbones has a worse prognosis than chondrosarcoma, making the distinction between these two malignant tumors the most important concern of the pathologist while dealing with a cartilage-forming tumor at this site [17]. Chondroblastic osteosarcoma is characterized by abundant hyaline chondroid matrix production associated with neoplastic bone formation represented by osteoid [6]. In the center of the lobules, chondrocytes show severe atypia while the peripheral areas are more cellular, containing spindle cells that surround tumor osteoid deposits [6, 15]. Thereby, the presence of a large amount of chondroid matrix makes the distinction between chondroblastic osteosarcoma and grade III chondrosarcoma difficult [18]. The identification of osteoid matrix deposits is the most determinant factor that leads to the diagnosis of chondroblastic osteosarcoma [15]. However, in grade III chondrosarcoma, endochondral ossification can lead to the formation of metaplastic bone in the form of trabecular eosinophilic matrix within the cartilage lobules that may be misinterpreted as osteoid deposit, leading to the diagnosis of chondroblastic osteosarcoma [16, 18]. In this case, the distinction between metaplastic bone and osteoid is the cornerstone of the differential diagnosis [8]. Osteoid appears generally in a lace-like configuration and occupies the lobules periphery where it is bordered by atypical cuboidal or spindled cells [8]. Besides, clinical and radiological features must be considered in the diagnosis evaluation. Osteosarcoma tends to occur in adolescents and young adults, while chondrosarcoma affects elderly populations [19]. Radiographic appearance of osseous matrix makes the diagnosis of chondrosarcoma unlikely [19]. Thus, diagnosis is based on a combination of clinical, pathologic, and radiologic findings.

Other entities can simulate grade III chondrosarcoma, including chondroid chordoma [6]. This tumor is composed of lobules and cords of large tumor cells with clear-to-light eosinophilic cytoplasm embedded in a large myxoid matrix that can closely mimic the architectural and cytological pattern of chondrosarcoma [6]. Although immunohistochemistry has no benefit in the differentiation of chondrosarcoma from other cartilage-forming tumors, it can help in the distinction between chondrosarcoma and other tumor entities [20]. Neoplastic chondrocytes express vimentin and S-100 [20], while chondroid chordoma shows strong immunoreactivity for brachyury, cytokeratin, and epithelial membrane antigen (EMA) [6].

### Therapeutic options

As for chondrosarcomas of other sites, surgical removal with wide surgical margin is considered the best therapeutic option for mandibular chondrosarcomas [8]. Radiotherapy and chemotherapy are not indicated in the primary treatment [4]. Distant tissue margins (˃ 2–3 cm) seem to enhance the prognosis and reduce risks of local recurrence [21]. Lymph node metastasis rarely occurs in jaw chondrosarcomas, and lymph node dissection is not necessary in all cases [4]. Distant metastasis have been rarely reported and is more likely to occur with high-grade or recurrent tumors. The most significant prognostic factors are size, location, histological grade, and resectability [4]. High-grade tumor differentiation and histologically positive margins are associated with poor prognosis [4]. Radiotherapy has an uncertain efficacy in the treatment of chondrosarcomas [8]. It is reserved as an adjuvant therapy for unresectable tumors and ones with positive margins or lymph node metastasis [12].

## Conclusion

The diagnosis of mandibular chondrosarcoma is challenging for pathologists owing to the rarity of the mandibular location of chondrosarcomas and the multiple entities that can mimic it histologically [21]. Early recognition followed by complete surgical resection are determinants for good prognosis [12]. In case of equivocal diagnosis, a multidisciplinary discussion involving maxillofacial surgeons, radiologists, pathologists, and oncologists should be carried out to optimize the patient’s care in terms of diagnosis and treatment [17].

## Data Availability

All data generated or analyzed during this study are included in this published article.
